# Host cell lectins ASGR1 and DC-SIGN jointly with TMEM106B confer ACE2 independence and imdevimab resistance to SARS-CoV-2 pseudovirus with spike mutation E484D

**DOI:** 10.1128/jvi.01230-24

**Published:** 2025-01-10

**Authors:** Prerna Arora, Lu Zhang, Inga Nehlmeier, Amy Kempf, Luise Graichen, Eike Kreitz, Anzhalika Sidarovich, Cheila Rocha, Sabine Gärtner, Michael Winkler, Sebastian Schulz, Hans-Martin Jäck, Markus Hoffmann, Stefan Pöhlmann

**Affiliations:** 1 Infection Biology Unit, German Primate Centre - Leibniz Institute for Primate Research 28361 , Göttingen, Germany; 2 Faculty of Biology and Psychology, Georg-August-University Göttingen 9375 , Göttingen, Germany; 3 Division of Molecular Immunology, Department of Internal Medicine 3, Friedrich-Alexander-University Erlangen-Nürnberg 138828 , Erlangen, Germany; Universite Laval, Laval, Quebec, Canada

**Keywords:** SARS-CoV-2, spike, entry, neutralization, lectin

## Abstract

The naturally occurring mutation E484D in the spike (S) protein of severe acute respiratory syndrome coronavirus 2 (SARS-CoV-2) can render viral entry ACE2 independent and imdevimab resistant. Here, we investigated whether the cellular proteins ASGR1, DC-SIGN, and TMEM106B, which interact with the viral S protein, can contribute to these processes. Employing S protein-pseudotyped particles, we found that expression of ASGR1 or DC-SIGN jointly with TMEM106B allowed for robust entry of mutant E484D into otherwise non-susceptible cells, while this effect was not observed upon separate expression of the single proteins and upon infection with SARS-CoV-2 wild type (WT). Furthermore, expression of ASGR1 or DC-SIGN conferred ACE2 independence and imdevimab resistance to entry of mutant E484D but not WT, and entry under those conditions was dependent on endogenous TMEM106B. These results suggest that engagement of certain cellular lectins can direct SARS-CoV-2 mutant E484D to an ACE2-independent, TMEM106B-dependent entry pathway that is not inhibited by imdevimab.

**IMPORTANCE:**

The interaction of severe acute respiratory syndrome coronavirus 2 (SARS-CoV-2) spike protein with the ACE2 receptor determines the viral cell tropism and is the key target of the neutralizing antibody response. Here, we show that SARS-CoV-2 with a single, naturally occurring mutation in the spike protein, E484D, can use the cellular lectins ASGR1 and DC-SIGN in conjunction with TMEM106B for ACE2-independent entry and evasion of therapeutic antibodies. These results suggest that engagement of cellular lectins might modulate target cell choice of SARS-CoV-2 and might allow evasion of certain neutralizing antibodies.

## INTRODUCTION

COVID-19 (coronavirus disease 2019) continues to threaten human health, particularly within at-risk groups, including the elderly and immunocompromised individuals. Severe acute respiratory syndrome coronavirus 2 (SARS-CoV-2), the causative agent of COVID-19, uses its spike (S) protein to enter target cells. Entry depends on the interaction of the surface unit of the S protein, termed S1, with the cellular receptor angiotensin-converting enzyme 2 (ACE2) and on the processing of the S protein by host cell proteases, which allows the transmembrane unit of the S protein, termed S2, to fuse the viral and a cellular membrane. Within the S1 subunit, a C-terminal receptor binding domain (RBD) engages ACE2, and the RBD/ACE2 interface is an important target for neutralizing antibodies induced upon infection and vaccination (1, 2).

In the course of the COVID-19 pandemic and the ensuing endemic, SARS-CoV-2 has constantly acquired mutations in the RBD and other portions of the S protein, which allow the virus to evade antibodies without critically compromising its ability to enter target cells. Residue E484 within the RBD is polymorphic, and substitutions like E484K and E484A allow for evasion of neutralizing antibodies (3). We previously reported that mutation E484D, which occurs naturally in a small fraction of patients, does not provide protection against neutralization by plasma from convalescent patients but is sufficient to allow for ACE2-independent entry into the hepatoma cell line Huh-7 (4). Furthermore, we found that Huh-7 cell entry of mutant E484D was highly resistant against the therapeutic monoclonal antibody imdevimab (REGN10987 [5, 6]), although the antibody efficiently inhibited entry into Vero kidney cells (4). The ability to enter cells in an ACE2-independent fashion might have significant implications for SARS-CoV-2 cell tropism and pathogenesis, and several receptors have been suggested to promote ACE2-independent entry, at least under conditions of overexpression (7). However, the molecular mechanism(s) responsible for ACE2-independence and imdevimab resistance of SARS-CoV-2 S protein mutant E484D remained unclear.

Gu et al. (8) reported that the cellular protein asialoglycoprotein receptor 1 (ASGR1) can facilitate ACE2-independent entry of SARS-CoV-2 wild type (WT), and a study by Yang et al. (9) indicated that ASGR1 promotes SARS-CoV-2 entry into Huh-7 hepatoma cells and primary hepatocytes. ASGR1 is a lectin that can recognize glycans on cellular and viral glycoproteins and can promote host cell entry of several viruses, including filoviruses (10, 11). Furthermore, Lempp et al. (12) provided evidence that engagement of the dendritic cell lectin DC-SIGN can reduce SARS-CoV-2 WT sensitivity to antibody-mediated neutralization. More recently, Baggen et al. (13) showed that TMEM106B, a 274 amino acid, comprising largely endo/lysosomal protein, binds to the RBD and allows for ACE2-independent entry into Huh-7 cells and several other cell lines. Notably, mutation E484D increased S protein binding to TMEM106B (13), but the role of TMEM106B in neutralization sensitivity of SARS-CoV-2 was not analyzed. Here, we investigated whether ASGR1, DC-SIGN, and TMEM106B can promote ACE2-independent and imdevimab-resistant cell entry of SARS-CoV-2 mutant E484D.

We found that directed expression of the lectins ASGR1 and DC-SIGN promotes ACE2-independent entry and imdevimab resistance and that this phenotype depends on coexpression of endogenous TMEM106B. Thus, certain cellular lectins might be able to re-route entry of SARS-CoV-2 E484D to an ACE2-independent pathway.

## RESULTS

### ACE2 independence of mutant E484D is associated with imdevimab and bebtelovimab resistance

Our previous study showed that ACE2-independent entry of mutant E484D into Huh-7 cells is associated with resistance to the neutralizing antibody imdevimab. We first sought to confirm and extend these findings, employing additional antibodies and cell lines. For this, we analyzed the human lung cell line NCI-H522 as well as the human liver cell line Li-7. NCI-H522 cells were previously shown by Puray-Chavez et al. (14) to allow for ACE2-independent entry of mutant E484D but not SARS-CoV-2, indicating that ACE2-independence is likely conferred by TMEM106B (13), while Gu et al. (8) found that SARS-CoV-2 WT entry into Li-7 cells was ACE2-independent and ASGR1-dependent (mutant E484D was not examined). We assessed entry into these cell lines and its inhibition by antibodies employing rhabdoviral pseudotypes bearing SARS-CoV-2 S protein, which adequately model host cell entry of authentic SARS-CoV-2 (1, 15, 16).

**Fig 1:**
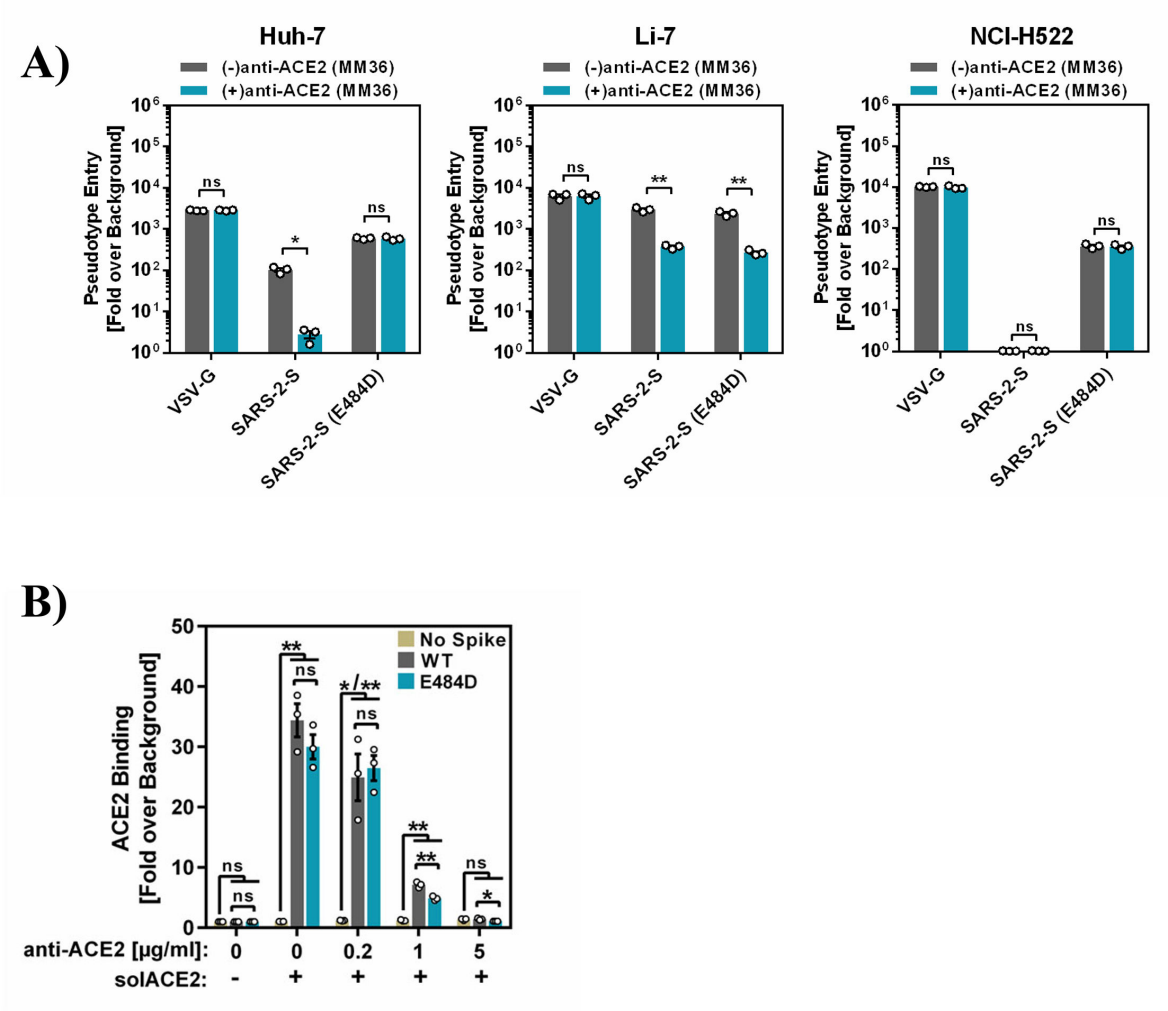
Huh-7 and NCI-H522 cells allow for ACE2-independent entry of mutant E484D. (**A**) ACE2 dependence of host cell entry. The indicated cell lines were grown in 96-well plates, incubated with ACE2 antibody (10108-MM36, Sino Biological) for 30 min and inoculated with pseudotypes bearing the indicated viral glycoproteins. Luciferase activity in cell lysates was quantified at 16–20 h postinoculation. The average of three independent experiments ± SEM is shown. Data were normalized against the assay background (i.e., particles bearing no glycoprotein, set as 1). Statistical significance was assessed by two-tailed Student’s *t*-test with Welch’s correction (*P* > 0.05, not significant [ns]; *P*  ≤  0.05, *; *P*  ≤  0.01, **; *P*  ≤  0.001, ***; not determined [nd]). (**B**) Mutation E484D does not allow for S protein binding to ACE2 in complex with an entry-inhibiting antibody. The indicated S proteins were transiently expressed in 293T cells and the cells incubated with soluble ACE2 preincubated with the indicated concentrations of anti-ACE2 antibody. ACE2 binding was detected by incubation with a secondary antibody and the cells analyzed by flow cytometry. Soluble ACE2 binding to cells transfected with empty plasmid served as control. The average ±SEM of three biological replicates conducted with unicate samples is shown. Data were normalized against the assay background (i.e., cells incubated with secondary antibody alone). Statistical significance was assessed by two-tailed Student’s *t*-test with Welch’s correction (*P* > 0.05, not significant [ns]; *P*  ≤  0.05, *; *P*  ≤  0.01, **).

We confirmed that particles pseudotyped with the S protein of SARS-CoV-2 mutant E484D (E484D_pp_) but not WT S protein (SARS-2-S_pp_) can enter Huh-7 liver cells in the presence of ACE2-blocking antibody (Fig. 1A) although both S proteins were unable to bind to ACE2 decorated with antibody (Fig. 1B). A similar observation was made for NCI-H522 lung cells, although SARS-2-S_pp_ were unable to appreciably enter these cells due to the previously documented absence of ACE2 expression (14). In contrast, entry of SARS-2-S_pp_ and E484D_pp_ into Li-7 liver cells was comparably reduced but not abrogated by anti-ACE2 antibody (Fig. 1A), potentially indicating an ACE2-independent pathway different from that operative in Huh-7 and NCI-H522 cells, although incomplete inhibition of ACE2-dependent entry instead of usage of an ACE2-independent entry pathway cannot be excluded.

**Fig 2:**
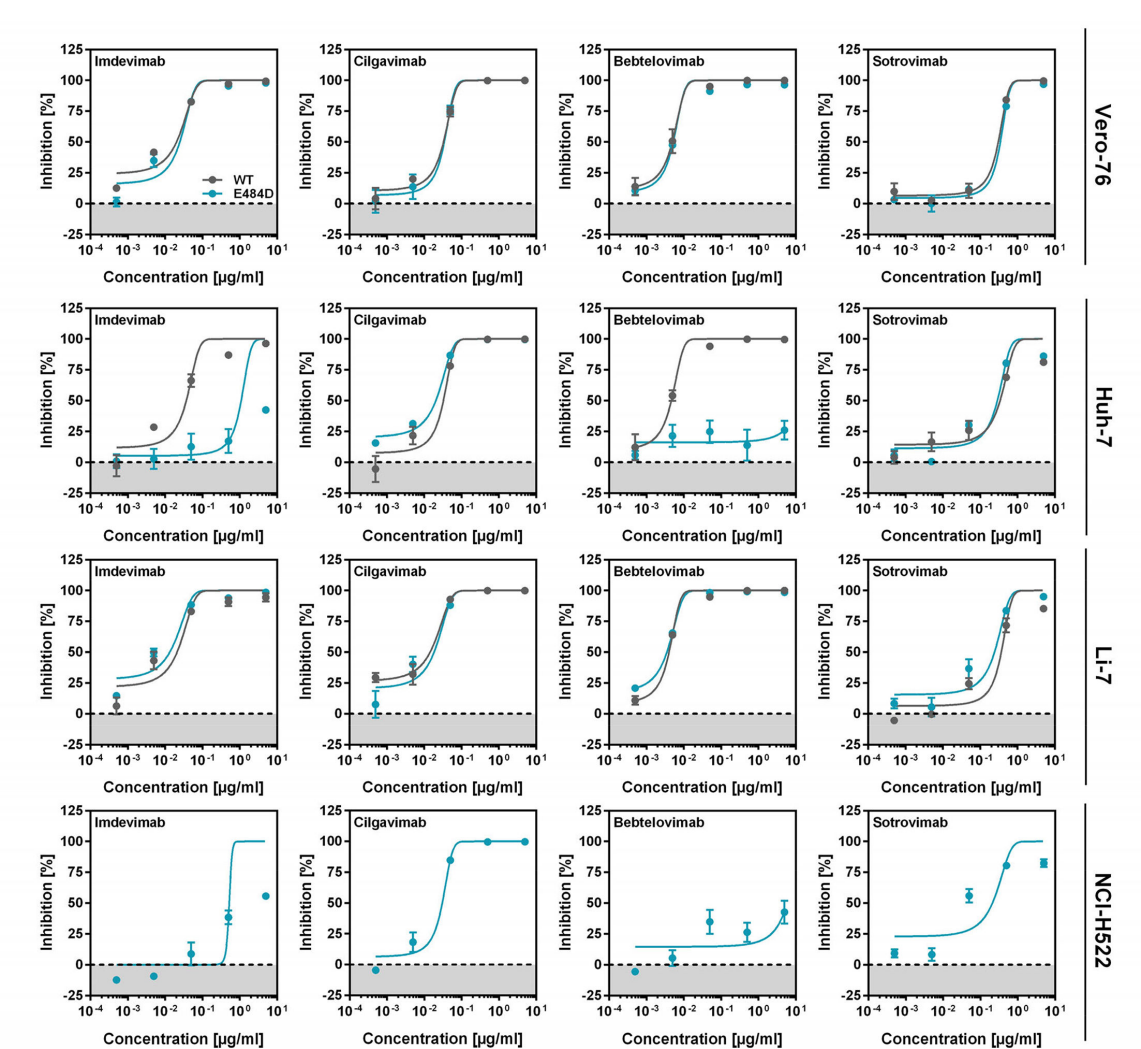
ACE2-independent entry correlates with resistance to imdevimab and bebtelovimab. Pseudotyped particles were preincubated with the indicated antibodies at the indicated concentrations for 1 h before addition to the indicated target cells. Luciferase activity in cell lysates was quantified at 16–20 h postinoculation. The average of three (two in case of NCI-H522 cells) independent experiments ± SEM is shown. Data were normalized against signals measured in the absence of antibody (= 0% inhibition). Curves were calculated using a non-linear regression model (variable slope).

We next analyzed whether Huh-7, Li-7, and NCI-H522 cell entry was associated with resistance against the antibodies bebtelovimab, cilgavimab, and sotrovimab. Imdevimab and the African green monkey kidney cell line Vero were used as controls since we previously found that imdevimab inhibits Vero cell entry of SARS-2-S_pp_ and E484D_pp_ with high and comparable efficiency, while E484D_pp_ entry into Huh-7 cells was largely imdevimab resistant (4). Since NCI-H522 cells do not express appreciable levels of ACE2 and are not susceptible to SARS-CoV-2 WT infection (14), only inhibition of E484D_pp_ entry into this cell line was examined. All antibodies tested blocked entry of SARS-2-S_pp_ and E484D_pp_ into Vero and Li-7 cells efficiently and comparably (Fig. 2). In contrast, E484D_pp_ entry into Huh-7 and NCI-H522 cells was largely insensitive to imdevimab and bebtelovimab (Fig. 2). These results show that ACE2-independent entry into Huh-7 and NCI-H522 cells of mutant E484D is associated with resistance against imdevimab and bebtelovimab (17), while entry of SARS-CoV-2 WT and mutant E484D into Li-7 cells is not.

### Coexpression of ASGR1 or DC-SIGN with TMEM106B promotes entry of mutant E484D

**Fig 3:**
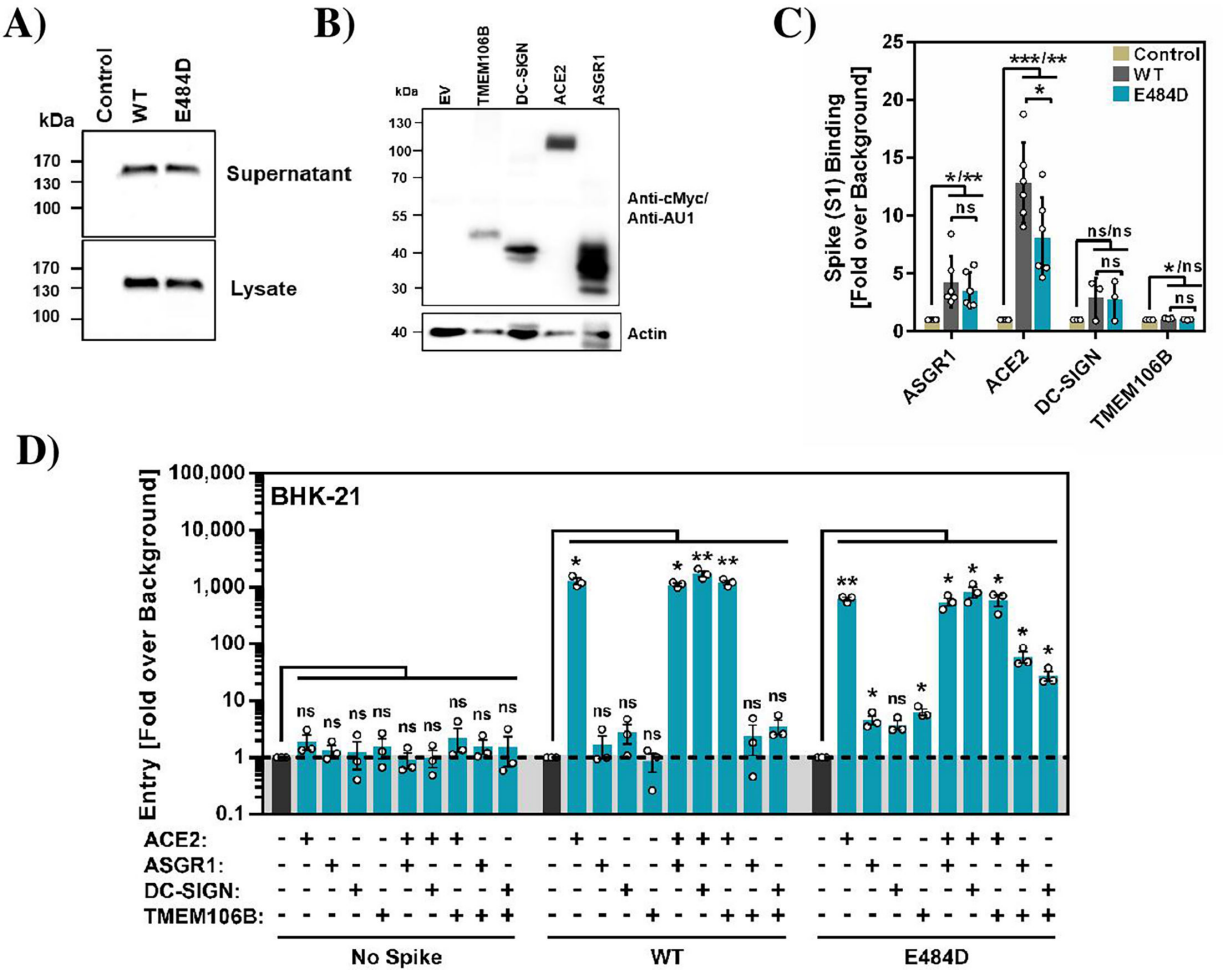
Directed expression of ASGR1/DC-SIGN jointly with TMEM106B allows for efficient entry into otherwise non-susceptible cells. (**A**) 293T cells were transfected to express the indicated soluble S proteins (S1 domain protein of B.1 [WT] and B.1 [E484D] S proteins fused to the Fc portion of human immunoglobulin G), and S protein levels in cell lysates and supernatants were analyzed by immunoblot using anti-Fc antibody. The results of a representative experiment are shown and were confirmed in two additional experiments. (**B**) 293T cells transfected to express ASGR1, DC-SIGN, TMEM106B, or ACE2 were analyzed by immunoblot using anti-c-Myc (DC-SIGN, TMEM106B, and ACE2) and anti-AU1 (ASGR1) antibody. The results of a representative experiment are shown and were confirmed in a separate experiment. (**C**) 293T cells transfected to express ASGR1, DC-SIGN, TMEM106B, or ACE2 were incubated with the indicated soluble S proteins, and S protein binding was analyzed by flow cytometry. Presented are the average (mean) data from three (DC-SIGN, TMEM106B) or six (ASGR1, ACE2) biological replicates (each conducted with single samples). Statistical significance was assessed by two-tailed Student’s *t*-test with Welch’s correction (*P* > 0.05, not significant [ns]; *P*  ≤  0.05, *; *P*  ≤  0.01, **). (**D**) BHK-21 cells were transfected with plasmids encoding ASGR1, DC-SIGN, TMEM106B, and ACE2 either alone or in combination and transduced with SARS-2-S_pp_ and E484D_pp_ followed by quantification of luciferase activity in cell lysates. Presented are the average (mean) data from three biological replicates (each conducted with four technical replicates), for which transduction was normalized against signals obtained from control-transfected cells inoculated with the respective pseudotyped particles (background, set as 1). Statistical significance was assessed by two-tailed Student’s *t*-test with Welch’s correction (*P* > 0.05, not significant [ns]; *P*  ≤  0.05, *; *P*  ≤  0.01, **).

Previous studies suggested that ASGR1, DC-SIGN, and TMEM106B can mediate ACE2-independent entry (8, 9, 13, 18), and we sought to determine whether their expression is sufficient to render cells susceptible. To address this question, we first asked whether the S proteins of WT and mutant E484D bind to ASGR1, DC-SIGN, and TMEM106B. For this, we fused the surface unit, S1, of the S protein to the Fc portion of human immunoglobulin (Fig. 3A) and analyzed binding to 293T cells transfected to express ASGR1, DC-SIGN, and TMEM106B (Fig. 3B). Cells transfected with empty vector (EV) served as negative control, while cells transfected with ACE2 encoding plasmid served as positive control. Both WT and E484D S proteins bound efficiently to ACE2 expressing cells, although binding of mutant E484D was slightly less efficient (Fig. 3C). Furthermore, WT S protein and mutant E484D bound comparably to DC-SIGN or ASGR1 transfected cells, but binding was less efficient as compared to ACE2 transfected cells. Finally, S protein binding to TMEM106B transfected cells was not detected (Fig. 3C), in keeping with TMEM106B mainly localizing to endo/lysosomes (13).

We next used BHK-21 cells to determine whether ASGR1, DC-SIGN, or TMEM106B exhibited receptor activity and whether coexpression of DC-SIGN and ASGR1 jointly with ACE2 or TMEM106B increased entry efficiency. BHK-21 cells were chosen because they are poorly susceptible to SARS-2-S_pp_ entry. We found that expression of ASGR1, DC-SIGN, or TMEM106B did not allow for appreciable SARS-2-S_pp_ and E484D_pp_ entry, although entry of E484D_pp_ was slightly elevated above background, and that coexpression of these proteins with ACE2 did not augment entry (Fig. 3D). In contrast, coexpression of TMEM106B with ASGR1 or DC-SIGN markedly increased entry of E484D_pp_ as compared to cells expressing either protein alone, and this effect was not observed for SARS-2-S_pp_ (Fig. 3D). Collectively, these results indicate that under the conditions chosen, expression of ASGR1 or DC-SIGN jointly with TMEM106B is sufficient for robust entry of mutant E484D, suggesting that TMEM106B may function as a receptor for mutant E484D that can only be used efficiently in the presence of lectins like ASGR1 and DC-SIGN.

### Directed expression of ASGR1 and DC-SIGN confers imdevimab resistance and ACE2 independence

**Fig 4:**
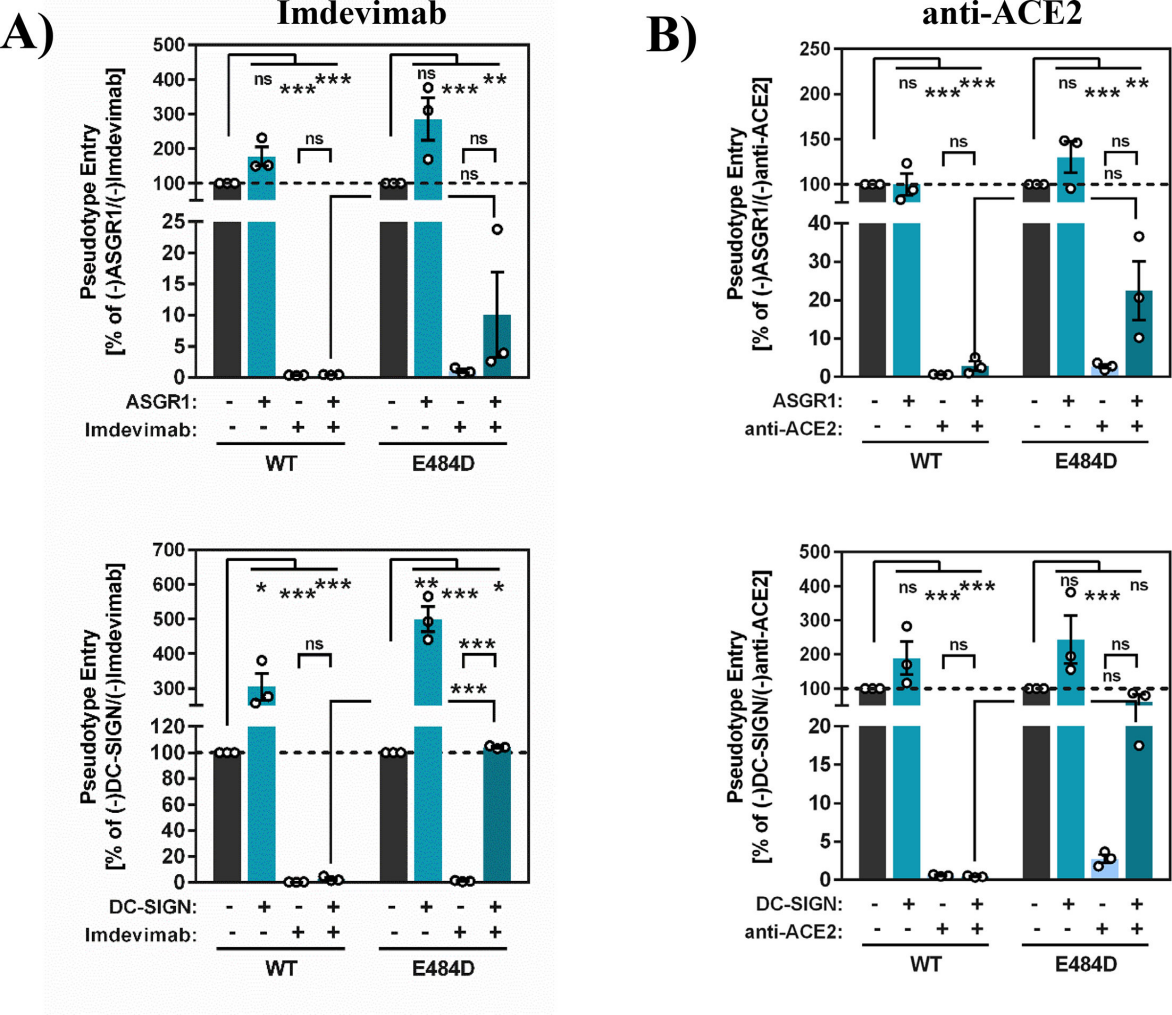
Directed expression of DC-SIGN or ASGR1 allows for imdevimab resistance and ACE2 independence of mutant E484D. For analysis of imdevimab resistance (A), 293T cells were transfected with ASGR1 or DC-SIGN encoding plasmids or empty plasmid as control and inoculated with pseudotypes that harbored the indicated S proteins and were pre-incubated with 1 µg/mL of imdevimab. Luciferase activities in cell lysates were quantified at 16–20 h postinoculation. For analysis of ACE2-independent entry (B), the experiment was conducted as described above, but cells were preincubated with 7 µg/mL anti-ACE2 antibody. Presented are the average (mean) data from three biological replicates (each conducted with four technical replicates), for which transduction was normalized against samples that did not contain antibody (= 100% pseudotype entry). Error bars indicate the SEM. Statistical significance was assessed by two-tailed Student’s *t*-test with Welch’s correction (*P* > 0.05, not significant [ns]; *P*  ≤  0.05, *; *P*  ≤  0.01, **; *P*  ≤  0.001, ***).

We next analyzed whether ASGR1 and DC-SIGN promote imdevimab resistance and ACE2 independence of mutant E484D. For this, we employed 293T cells, which express endogenous ACE2 and TMEM106B (see below) but are negative for DC-SIGN and ASGR1. Directed expression of both ASGR1 and DC-SIGN conferred little or no resistance of SARS-2-S_pp_ to imdevimab (Fig. 4, panel A) or anti-ACE2 antibody (Fig. 4, panel B). In contrast, expression of ASGR1 and particularly DC-SIGN allowed for robust entry of E484D_pp_ in the presence of imdevimab or anti-ACE2 antibody (Fig. 4), indicating that ASGR1 and DC-SIGN and potentially other cellular lectins can promote imdevimab resistance and ACE2 independence of SARS-CoV-2 mutant E484D.

**Fig 5:**
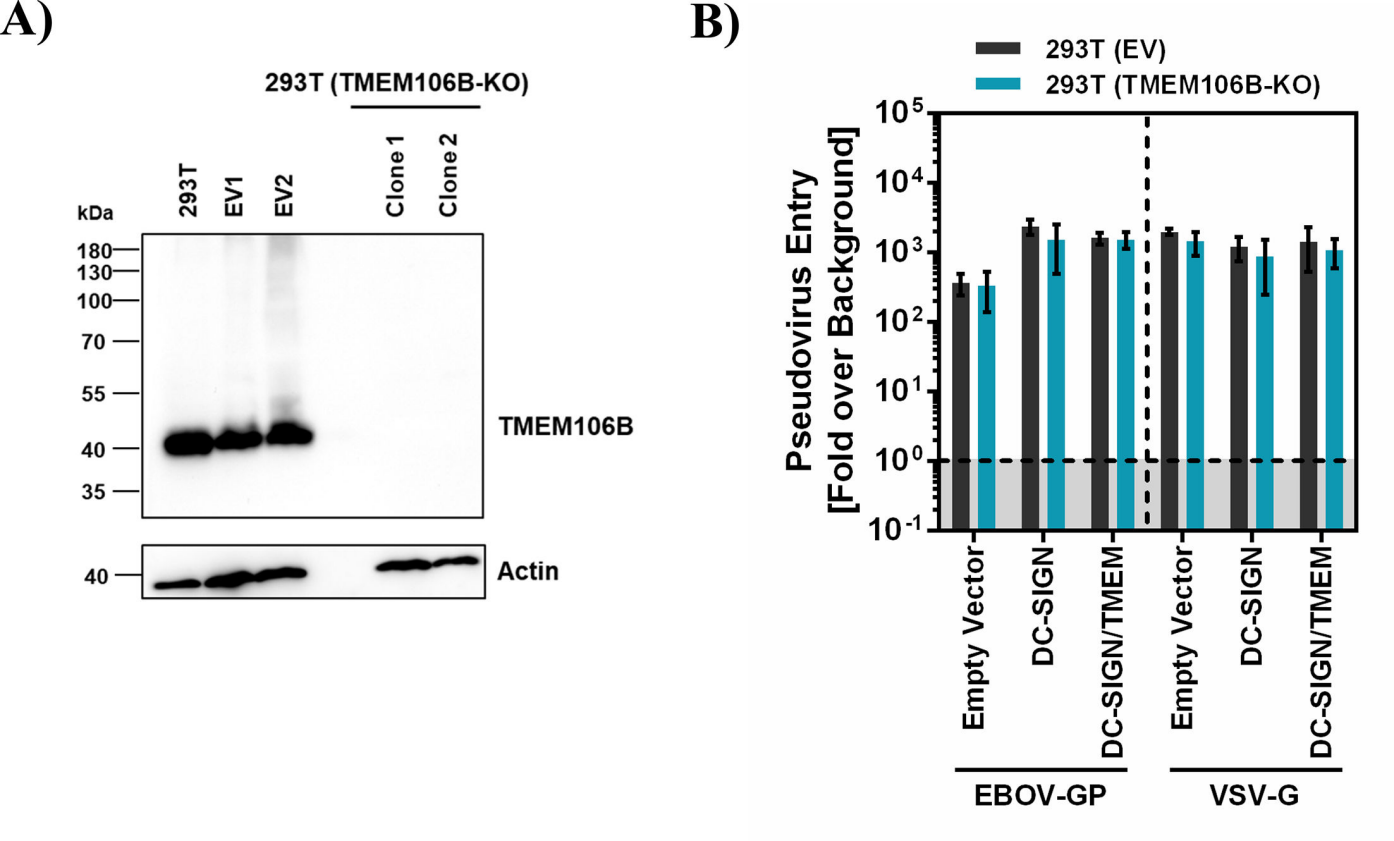
KO of TMEM106B in 293T cells. (**A**) TMEM106B expression in 293T WT and TMEM106B KO cells. 293T cells were stably transfected with EV (control) or vector encoding TMEM106B specific guide RNAs followed by antibiotic selection and single cell cloning. Parental (293T), two clones of control 293T cells (EV1, EV2) and two clones of TMEM106B KO cell lines (Clone 1, Clone 2) were lysed and analyzed for TMEM106B expression by immunoblot, using TMEM106B specific antibody. Similar results were obtained in two separate experiments. (**B**) Control or TMEM106B KO 293T cells transfected with EV or DC-SIGN plasmid or cotransfected with DC-SIGN and TMEM106B plasmids were transduced with particles pseudotyped with EBOV-GP or VSV-G. At 16–20 h postinoculation, transduction efficiency was analyzed by quantifying luciferase activity in cell lysates. Presented are the results of a representative experiment conducted with technical quadruplicates; error bars indicate SD. Transduction was normalized against signals obtained for control particles bearing no viral glycoprotein (background, set as 1). Similar results were obtained in two separate experiments.

**Fig 6:**
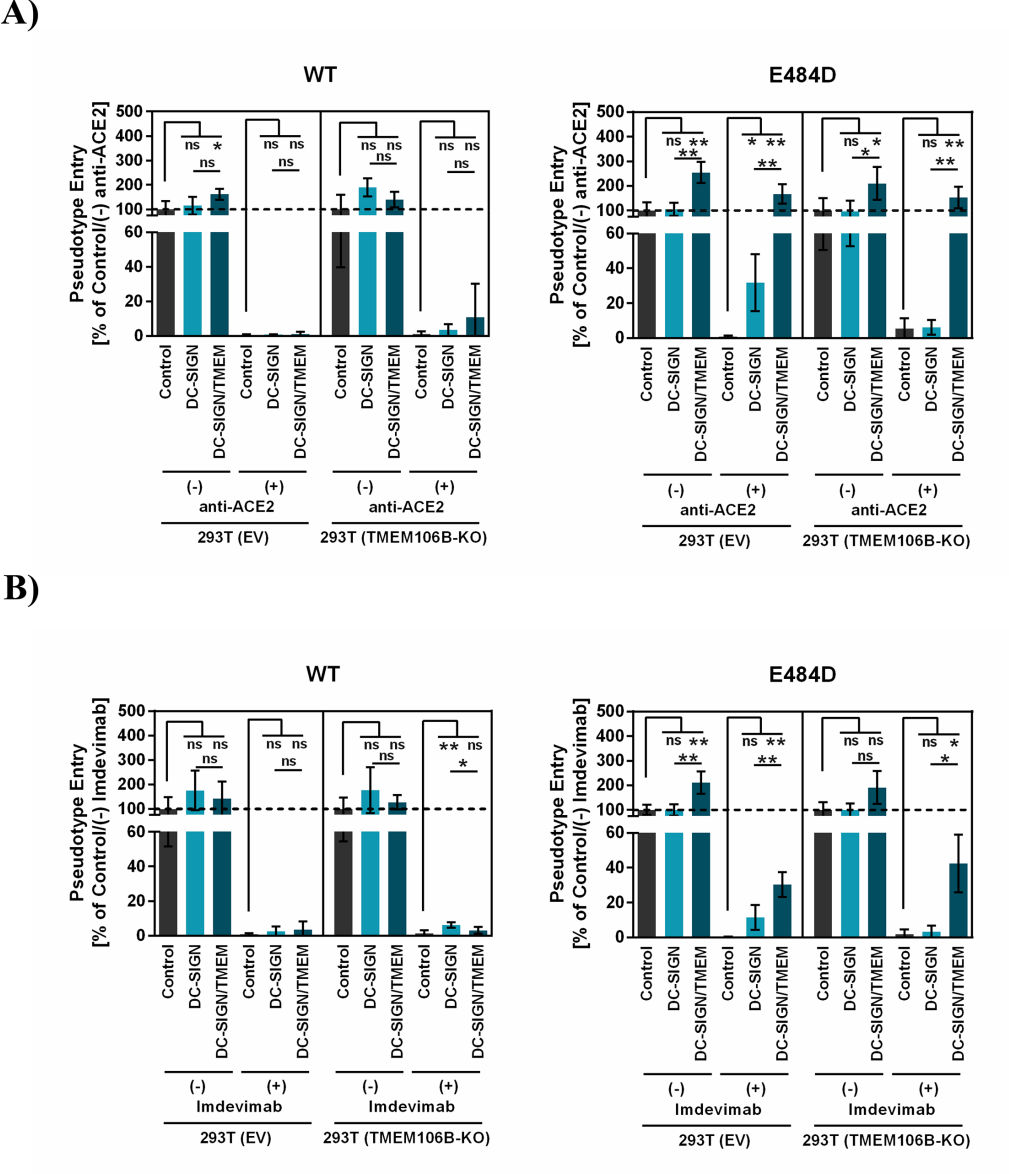
Endogenous expression of TMEM106B is required for DC-SIGN-dependent imdevimab resistance and ACE2 independence of mutant E484D. For analysis of ACE2-independent entry (A), 293T control (EV) or TMEM106B KO cells were transiently transfected with EV (control), or DC-SIGN plasmid or cotransfected with DC-SIGN and TMEM106B plasmids, incubated with 7 µg/mL of anti-ACE2 antibody and inoculated with pseudotypes bearing the indicated S proteins followed by quantification of luciferase activities in cell lysates at 16–20 h postinoculation. For analysis of imdevimab resistance (B), the experiment was conducted as described above, but pseudotyped particles were preincubated with 1 µg/mL imdevimab. Presented are the results from a representative experiment carried out with four technical replicates; transduction was normalized against samples that did not contain antibody. Error bars indicate SD, similar results were obtained in a separate experiment. Statistical significance was assessed by two-tailed Student’s *t*-test with Welch’s correction (*P* > 0.05, not significant [ns]; *P*  ≤  0.05, *; *P*  ≤  0.01, **; *P*  ≤  0.001, ***).

The 293T cell line expresses endogenous TMEM106B (Fig. 5A), and TMEM106B might be required for imdevimab resistance and ACE2-independent entry in lectin expressing 293T cells. To address this question, we employed CRISPR/Cas9 to knock out TMEM106B expression in 293T cells and repeated the above-described infection experiment using DC-SIGN as model lectin. Immunoblot analysis confirmed knockout (KO) of TMEM106B (Fig. 5A), and directed expression of DC-SIGN in both 293T control and TMEM106B KO cells increased entry of Ebola virus glycoprotein (EBOV-GP)_pp_ with similar efficiency but had no effect on vesicular stomatitis virus glycoprotein (VSV-G)_pp_ entry (Fig. 5B), indicating that TMEM106B KO did not interfere with lectin-mediated enhancement of infection (Fig. 5B). Further, inoculation of 293T cells with S protein bearing pseudotypes confirmed that DC-SIGN efficiently rescues E484D_pp_ but not SARS-2-S_pp_ from inhibition by imdevimab and anti-ACE2 antibody (Fig. 6). Finally, DC-SIGN-dependent rescue of E484D_pp_ from antibody-mediated inhibition was not observed in TMEM106B KO cells and was reinstalled upon directed expression of TMEM106B (Fig. 6). Thus, DC-SIGN jointly with TMEM106B but not either protein alone can allow for imdevimab resistance and ACE2 independence of SARS-CoV-2 mutant E484D in the cellular system chosen.

### Evidence that endogenous lectins contribute to ACE2-independent Huh-7 cell entry of mutant E484D

**Fig 7:**
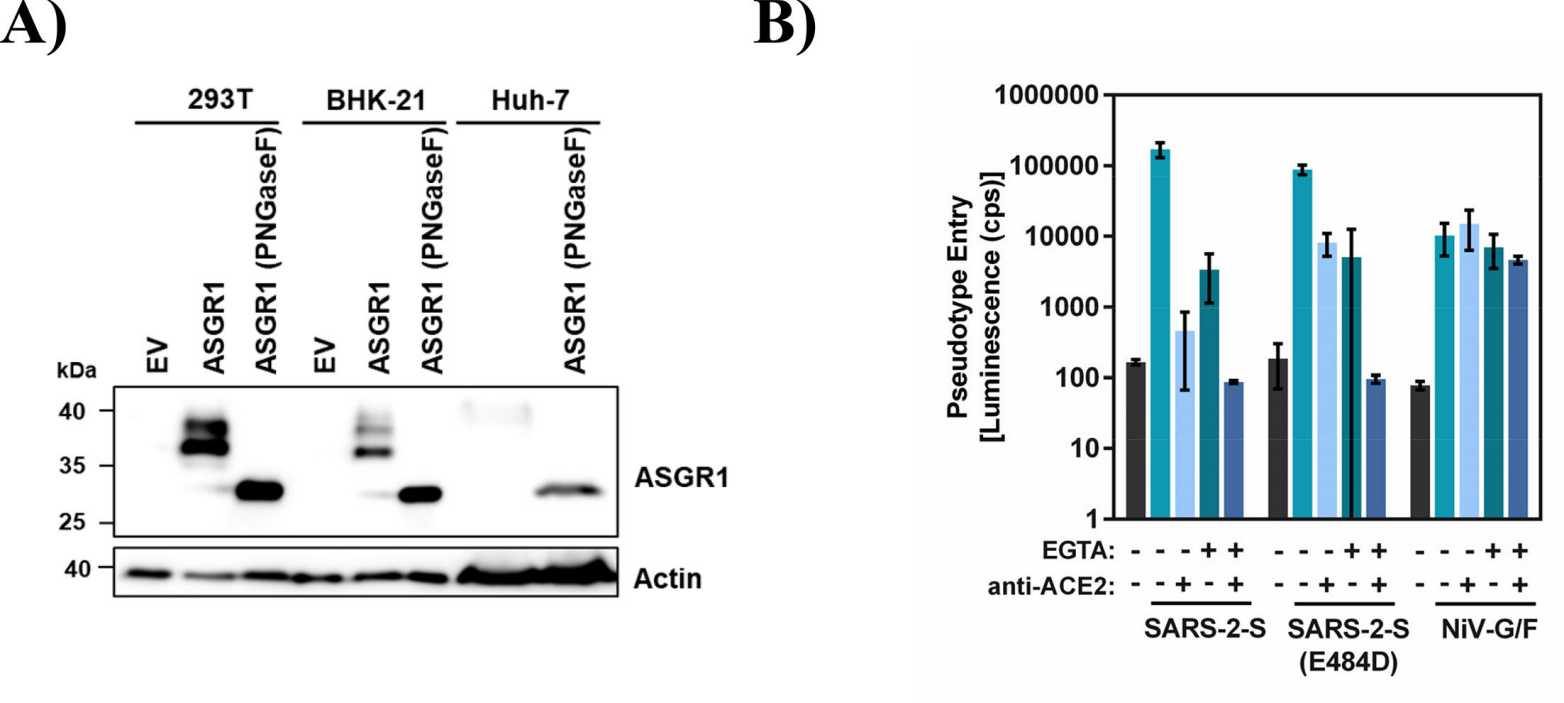
EGTA blocks ACE2-independent entry into Huh-7 cells. (**A**) Expression and N-glycosylation of ASGR1 in Huh-7 cells. Untransfected Huh-7 cells and 293T and BHK21 cells transfected with EV or ASGR1 encoding vector were control treated or treated with PNGaseF and analyzed for ASGR1 expression by immunoblot. Expression of β-actin served as loading control. Similar results were obtained in two (all cell lines) to four (293T, BHK21 cells) independent experiments. (**B**) Huh-7 cells were preincubated with medium alone (control) or medium containing anti-ACE2 antibody, EGTA, or anti-ACE2 antibody combined with EGTA for 1 h followed by inoculation with pseudotypes bearing the indicated S proteins. Luciferase activity in cell lysates was quantified at 16–20 h postinoculation. The results ± SD of a single experiment performed with technical quadruplicates are shown. Similar results were obtained in a separate experiment.

Evasion of anti-ACE2 antibody by E484D_pp_ was observed with cells transfected to express ASGR1 or DC-SIGN. We sought to obtain initial evidence that endogenous lectin expression can contribute to ACE2-independent entry. Since we are not aware of cell lines that express robust levels of endogenous DC-SIGN, we focused on Huh-7 cells, which express endogenous ASGR1 (Fig. 7A) and TMEM106B (19). Endogenous ASGR1 in Huh-7 cells migrated slightly slower in SDS-PAGE than transfected ASGR1 in BHK21 and 293T cells, due to differences in N-glycosylation as revealed by PNGaseF digest (Fig. 7A). Unfortunately, we failed to reproducibly and specifically knock down or knock out ASGR1 expression in Huh-7 cells. Therefore, we used EGTA to determine a potential contribution of C-type lectins like ASGR1 to ACE2-independent Huh-7 cell entry. The chelator EGTA removes Ca^++^ ions that are required for the structural integrity of C-type lectins and thereby blocks lectin binding to ligands. Treatment of Huh-7 cells with anti-ACE2 antibody reduced SARS-2-S_pp_ entry close to background levels, while inhibition of E484D_pp_ was less efficient, as expected (Fig. 7B). Furthermore, both SARS-2-S_pp_ and E484D_pp_ were comparably inhibited by EGTA, and inhibition of SARS-2-S_pp_ by EGTA was less potent as compared to that observed with anti-ACE2 antibody. Finally, combination treatment with anti-ACE2 antibody and EGTA reduced entry of SARS-2-S_pp_ and E484D_pp_ to background levels, and these effects were specific since neither EGTA nor anti-ACE2 antibody appreciably interfered with entry driven by the Nipah virus F and G proteins (Fig. 7B). The observation that E484D_pp_ entry into Huh-7 cells pretreated with anti-ACE2 antibody was robust but was reduced to the background level by EGTA is compatible with the concept that C-type lectins, potentially ASGR1, promote ACE2-independent entry of mutant E484D into Huh-7 cells.

## DISCUSSION

The present data jointly with previous results (4, 13, 14, 19) reveal a pathway for ACE2-independent entry. The pathway is employed by mutant E484D but not WT for entry into Huh-7 liver and NCI-H522 lung cell lines and is associated with resistance against imdevimab and bebtelovimab. Further, expression of DC-SIGN and ASGR1 can direct mutant E484D to the ACE2-independent pathway, which is dependent on TMEM106B.

The TMEM106B-dependent entry route mainly used by mutant E484D might not be the only one since Gu et al. (8) reported ACE2-independent NCI-H520 cell entry of SARS-CoV-2 WT. Furthermore, Gu et al. (8) found that ACE2-independent entry of SARS-CoV-2 WT into Li-7 cells was robust, as indicated by marked resistance against antibodies targeting the S protein/ACE2 interface, while the ACE2 antibody used in the present study blocked entry by roughly 80%. Use of different antibodies to examine ACE2-independence might account for this difference.

The finding that ACE2-independent entry of mutant E484D was associated with resistance against imdevimab and bebtelovimab is in keeping with these antibodies recognizing very similar epitopes (17), which are apparently dispensable for ACE2-independent entry. Our findings are also in keeping with a previous study reporting that directed DC-SIGN expression in 293T cells reduces SARS-CoV-2 WT neutralization by imdevimab and other antibodies targeting the receptor binding motif (12). However, we observed this phenotype mainly for mutant E484D, potentially because of reduced DC-SIGN expression levels on target cells used in the present as compared to the previous study. ACE2-independent entry of mutant E484D and imdevimab evasion upon directed expression of DC-SIGN was dependent on endogenous TMEM106B, which binds to the RBD, and binding is augmented by mutation E484D (13). These findings suggest that DC-SIGN and ASGR1 concentrate WT and mutant E484D at the cell surface, which then mainly enables mutant E484D to efficiently engage TMEM106B in a fashion that is not inhibited by imdevimab and bebtelovimab. Whether this mechanism is operative *in vivo* and impacts cell tropism and antibody evasion remains to be determined, and a recent study employing murine models suggested that availability of ACE2 remains critical for establishment of infection despite the presence of mutation E484D (20). However, it is conceivable that, for instance, DC-SIGN on alveolar macrophages (21, 22) promotes SARS-CoV-2 entry into these cells, which express only low levels of ACE2 (23, 24) and allow for viral persistence (25). Finally, it is noteworthy that of all naturally occurring amino acid substitutions at position 484, only E484D has been associated with ACE2 independence (4), but viruses harboring E484D rarely arise in patients. The underlying reason is unclear, considering that a SARS-CoV-2 variant harboring E484D and other mutations in the S protein replicates efficiently and causes disease in a mouse model (26).

Our study has limitations, including the use of pseudotyped viruses instead of replication competent SARS-CoV-2 and the analyses of lectins mainly upon directed but not endogenous expression, although it is noteworthy that EGTA, which inhibits ligand binding to ASGR1, reduced ACE2-independent entry into Huh-7 cells to background levels. Despite these limitations, our results point towards a cooperative role of lectins and TMEM106B in ACE2-independent entry and antibody evasion that deserves further investigation.

## MATERIALS AND METHODS

### Cell cultures

All cell lines used in this study were maintained at 37°C and 5% CO_2_ in a humidified atmosphere. 293T (human, female, kidney; ACC-635, DSMZ, RRID:CVCL_0063), Huh-7 (human, male, liver; JCRB0403, JRCB; RRID:CVCL_0336, kindly provided by Thomas Pietschmann), BHK-21 (Syrian hamster, kidney; ATCC Cat# CCL-10; RRID:CVCL_1915, kindly provided by Georg Herrler), Vero76 (African green monkey, female, kidney; CRL-1586, ATCC; RRID:CVCL_0574, kindly provided by Andrea Maisner), NCI-H522 (human, male, lung, ATCC: CRL-5810, RRID:CVCL_1567), and Li-7 (human, male, liver, RCB1941, RIKEN BRC; RRID:CVCL_3840) cells were maintained in Dulbecco’s modified Eagle medium (PAN-Biotech). All media were supplemented with 10% fetal bovine serum (FBS, Biochrom), 100 U/mL of penicillin and 0.1 mg/mL of streptomycin (PAN-Biotech). Cell lines were validated by short tandem repeat typing, sequencing of part of the cytochrome c oxidase gene, microscopic examination, and/or according to their growth characteristics. Cell lines were regularly analyzed for mycoplasma contamination. Transfection of cells was carried out by calcium phosphate precipitation (293T) or using Lipofectamine 2000 (BHK-21; Thermo Fisher Scientific).

### Plasmids

Expression plasmids for ACE2 (27), ASGR1 (28), DC-SIGN (28), VSV glycoprotein (VSV-G) (1, 29), the glycoprotein of Ebola virus (EBOV-GP) (30, 31), the glycoprotein and fusion protein of Nipah virus (NiV-G/F) (32), the S proteins of WT SARS-CoV-2 (PANGO lineage B.1; codon-optimized, C-terminal truncation of the last 18 amino acids) and mutant E484D have been described elsewhere (1, 4, 32). For construction of soluble S proteins consisting of the S1 subunit of SARS-2-S WT or mutant E484D, the corresponding genetic information (coding for S protein residues 1 to 677) was PCR-amplified and inserted into the pCG1-Fc expression plasmid (33) (kindly provided by Georg Herrler) using BamHI and XbaI restriction sites. Integrity of all PCR-amplified sequences was confirmed by sequencing using a commercial service (Seqlab).

### Production of pseudotyped particles

For production of pseudotyped particles, a replication-deficient VSV-derived vector was employed, VSV^∗^ΔG-FLuc, that was kindly provided by Gert Zimmer (34). In the VSV∗ΔG-FLuc genome, the genetic information for VSV-G has been deleted, and the vector codes for two reporter proteins, enhanced green fluorescent protein and firefly luciferase (FLuc), instead. Pseudotyping of VSV was carried out essentially as described (35). In brief, 293T cells were transfected with expression plasmids encoding S protein, EBOV-GP, NiV-H/F, VSV-G, or empty plasmid (control) and, after a 24-h incubation, inoculated with VSV^∗^ΔG-FLuc at a multiplicity of infection of 3. At 1–2 h postinoculation, cells were washed with phosphate-buffered saline (PBS) and incubated in the presence of anti-VSV-G antibody (culture supernatant from I1-hybridoma cells; ATCC no. CRL-2700; except for cells expressing VSV-G, which received only medium). At 16–20 h postinoculation, the culture supernatants were collected, clarified from cellular debris using a 0.45-µm filter, aliquoted, and stored at −80°C until further use.

### Binding of S protein to ASGR1 and ACE2

In order to test binding of S proteins to ASGR1 and ACE2, 293T cells were seeded in 6-well plates and transfected with plasmids encoding ACE2 and ASGR1 by calcium-phosphate precipitation. Cells transfected with empty plasmid served as a negative control. At 24 h posttransfection, the medium was replaced with fresh culture medium. At 48 h posttransfection, the culture medium was removed, and cells were resuspended in PBS containing Ca^++^ and Mg^++^ ions, and 5% FBS (PBS-F) and transferred into 1.5-mL reaction tubes before being pelleted by centrifugation. All centrifugation steps were carried out at 600 × *g* for 5 min, 4°C. Subsequently, the supernatant was aspirated and the cells washed with PBS-F. Next, cell pellets were resuspended in 100-µL PBS-F containing comparable amounts (as judged by immunoblot) of soluble SARS-2-S (WT) and SARS-2-S (E484D) fused to the Fc portion of human immunoglobulin G and incubated for 60 min at 4°C. Following incubation, cells were washed, resuspended in 100-µL PBS-F containing anti-human AlexaFluor-488-conjugated antibody (1:250; Thermo Fisher Scientific), and incubated again for 60 min at 4°C. Finally, the cells were washed with PBS-F, fixed by incubation in 2% paraformaldehyde solution for 30 min at RT, washed again, and resuspended in 200 µL PBS-F before being subjected to flow cytometric analysis using an ID7000 Spectral Analyser (Sony) and ID7000 software.

### Analysis of S protein binding to soluble ACE2 by flow cytometry

In order to test S protein binding to soluble ACE2 in the presence and absence of anti-ACE2 antibody by flow cytometry, 293T cells were transfected with EV or plasmid encoding SARS-2-S WT or E484D mutant. Medium was replaced by fresh culture medium at 24 h posttransfection, and cells were harvested at 48 h posttransfection. For this, cells were washed with PBS, resuspended in PBS-F, and pelleted by centrifugation at 600 × *g* for 5 min, 4°C. Next, cells were resuspended in PBS-F supplemented with soluble ACE2-Fc (1 µg/mL; Acro Biosystems), which was pre-incubated for 30 min in the presence of different concentrations of anti-ACE2 antibody (0, 0.2, 1, and 5 µg/mL; SinoBiological). Following an incubation period of 1 h at 4°C on a rotating disc (IKA), cells were washed thrice, pelleted, resuspended in PBS-F containing anti-human AlexaFluor-488-conjugated antibody (1:250; Thermo Fisher Scientific), and incubated again for 60 min at 4°C. Finally, the cells were washed thrice with PBS-F and fixed in 2% paraformaldehyde overnight at 4°C. Next day, cells were washed, resuspended in PBS-F, and subjected to flow cytometric analysis using an ID7000 Spectral Analyser (Sony). Data were further analyzed using the ID7000 analysis software.

### Immunoblot

To investigate S1-Fc protein levels in cell lysates and supernatants, 293T cells were transfected to express soluble S proteins (S1 domain fused to the Fc portion of immunoglobulin). At 24 h posttransfection, medium was removed, and fresh culture medium supplemented with 1% FBS was added. To detect S proteins in cell lysates, cells were lysed at 72 h posttransfection by incubation in 2× SDS-sample buffer (0.03 M Tris-HCl, 10% glycerol, 2% SDS, 5% beta-mercaptoethanol, 0.2% bromophenol blue, and 1-mM EDTA). For analysis of S protein levels in supernatants, supernatants were harvested at 48 and 72 h posttransfection (after the first harvest at 48 h, cells received fresh medium without FBS). The supernatants of both harvests were combined and concentrated (20×) using Vivaspin protein concentrator columns (50 kDa molecular weight cut-off) at 4,000 × g. Concentrated soluble S1-Fc proteins were aliquoted and stored at −80°C until further use. For immunoblot analysis, equal volumes of concentrated supernatants and 2× SDS-sample buffer were mixed and incubated at 95°C for 10 min, before being subjected to SDS-PAGE. For analysis of ASGR1 expression in 293T, BHK-21, and Huh-7 cells, 293T and BHK-21 cells were transfected with EV or ASGR1 encoding plasmid, while Huh-7 cells remained untransfected. At 48 h posttransfection, cells were lysed by incubation in 2× SDS-sample buffer at 95°C for 10 min. For analysis of expression of TMEM106B, DC-SIGN, ACE2, and ASGR1 in transfected 293T cells, cells were transfected with the respective plasmids and harvested in 2× SDS-sample buffer at 48 h posttransfection.

For all immunoblot analyses, lysates were blotted onto nitrocellulose membranes (Hartenstein) and membranes blocked for 30 min in PBS-T (PBS with 0.5% Tween 20) containing 5% skim milk. After blocking, membranes were incubated with horseradish peroxidase (HRP)-conjugated anti-human antibody (1:5,000, Dianova) for detection of S1-Fc protein. For detection of ASGR1 expression in lysates of Huh-7 cells or lysates of transfected 293T and BHK-21 cells, a mouse anti-ASGR primary antibody (1:500, Santa Cruz) was used in combination with goat anti-mouse secondary antibody (1:5000, Dianova). For detection of TMEM106B, DC-SIGN, ACE2, or ASGR1, a mixture of antibodies against the cMyc (undiluted supernatant of clone 9E10 hybridoma, ATCC) and AU1 (1:500 diluted, Covance,) antigenic tags was used; HRP-conjugated goat anti-mouse antibody (diluted in 1:5000 in 5% skim milk, Dianova) was employed as secondary antibody. For analysis of TMEM106B expression in 293T WT and KO cells, an anti-TMEM106B primary antibody (1:2000, Cell Signalling,) and an anti-rabbit (1:2500, Dianova) secondary antibody were used. As loading control, ß-actin expression was detected using anti-ß-actin primary antibody (1:1000, Sigma-Aldrich) and HRP-conjugated goat anti-rabbit secondary antibody (1:5,000, Dianova). After antibody incubation, blots were washed three times for 5 min with PBS-T. Immunoblots were developed using a self-made chemiluminescence solution (0.1 M Tris-HCl [pH 8.6], 250-µg/mL luminol, 0.1-mg/mL para-hydroxycoumaric acid, and 0.3% hydrogen peroxide) in combination with the ChemoCam imaging system and the ChemoStar Professional software (Intas Science Imaging Instruments)

### Construction of 293T TMEM106B KO cell lines

293T TMEM106B KO cell lines were generated using the CRISPR-Cas9 system. For this, guide RNAs for two separate targets in the *tmem106b* gene were selected (gRNA-1: GAGTCACATCTGAAAACATGAGG and gRNA-2: GGAACAGGAAGAATTCCTAGGGG; the protospacer adjacent motif [PAM] is underlined) and the respective sequences (without PAM) inserted into the pLentiCRISPR v2 vector (Addgene plasmid #52961). The resulting plasmids were transfected into 293T cells plated in 6-well dishes jointly with plasmids encoding VSV-G and HIV-gag-pol. Supernatants were harvested at 48 h posttransfection and used for transduction of freshly seeded 293T cells followed by puromycin selection (pLentiCRISPR v2 encodes a puromycin resistance gene). Finally, single cells were seeded in 96-well plates and clonal colonies expanded and analyzed by immunoblot.

### Transduction of target cells

At 24 h prior to transduction, target cells were seeded in 96-well plates. The following experimental designs were chosen: (i) In order to investigate a potential receptor function of ASGR1 and DC-SIGN, 293T or BHK-21 cells, which were transfected to express these proteins, were inoculated with equal volumes of pseudotype particles. Particles bearing EBOV-GP, VSV-G, or no viral surface protein served as controls. (ii) In order to assess ACE2-independent entry into cell lines, Huh-7, Li-7, and NCI-H522 cells were preincubated (30 min, 37°C) with medium containing 7 µg/mL of anti-ACE2 neutralizing mouse monoclonal antibody (10108-MM36, Sino Biological), before addition of equal volumes of pseudotyped particles. Cells incubated with medium without antibody served as controls. (iii) In order to determine ACE2-independence following directed expression of ASGR1 or DC-SIGN, 293T cells transfected to express ASGR1, DC-SIGN, or empty plasmid were incubated with anti-ACE2 antibody at 7 µg/mL for 30 min and subsequently inoculated with pseudotypes bearing SARS-2-S WT or mutant E484D. (iv) In order to determine imdevimab resistance following directed expression of ASGR1 or DC-SIGN, pseudotype particles were incubated with imdevimab at 1 µg/mL for 30 min and subsequently added to 293T cells transfected to express ASGR1, DC-SIGN, or empty plasmid. (v) In order to determine the contribution of ASGR1 (and related proteins) to ACE2-independent entry into Huh-7 cells, the cells were preincubated with medium containing anti-ACE2 antibody (7 µg/mL, 10108-MM36, Sino Biological), EGTA (5 mM), anti-ACE2 antibody combined with EGTA, or medium only for 1 h followed by inoculation with equal volumes of pseudotypes bearing SARS-2-S WT, SARS-2-S E484D, or NiV F/G. For all experiments, transduction efficiency was analyzed at 16–20 h postinoculation by measuring FLuc activity in cell lysates. For this, the culture medium was removed, and the cells were lysed for 30–60 min at RT with PBS containing 0.5% Triton X-100 (Carl Roth). Lysates were analyzed in white 96-well plates for luciferase activity, using a commercial substrate (Beetle-Juice, PJK GmbH) and a Hidex Sense plate luminometer (Hidex).

### Data analysis

Data ware analyzed using Microsoft Excel (as part of the Microsoft Office software package, version 2019, Microsoft Corporation) and GraphPad Prism 6 version 6.07 (GraphPad Software). Statistical significance was assessed by two-tailed Student’s *t*-test with Welch’s correction) (*P* > 0.05, not significant [ns]; *P*  ≤  0.05, *; *P*  ≤  0.01, **; *P*  ≤  0.001, ***).

## Data Availability

All data relevant to the study are shown in the paper.

## References

[1] Hoffmann M, Kleine-Weber H, Schroeder S, Krüger N, Herrler T, Erichsen S, Schiergens TS, Herrler G, Wu N-H, Nitsche A, Müller MA, Drosten C, Pöhlmann S. 2020. SARS-CoV-2 cell entry depends on ACE2 and TMPRSS2 and is blocked by a clinically proven protease inhibitor. Cell 181:271–280. doi:10.1016/j.cell.2020.02.052 32142651 PMC7102627

[2] Zhou P, Yang X-L, Wang X-G, Hu B, Zhang L, Zhang W, Si H-R, Zhu Y, Li B, Huang C-L, et al.. 2020. A pneumonia outbreak associated with a new coronavirus of probable bat origin. Nature 579:270–273. doi:10.1038/s41586-020-2012-7 32015507 PMC7095418

[3] Cao Y, Wang J, Jian F, Xiao T, Song W, Yisimayi A, Huang W, Li Q, Wang P, An R, et al.. 2022. Omicron escapes the majority of existing SARS-CoV-2 neutralizing antibodies. Nature 602:657–663. doi:10.1038/s41586-021-04385-3 35016194 PMC8866119

[4] Hoffmann M, Sidarovich A, Arora P, Krüger N, Nehlmeier I, Kempf A, Graichen L, Winkler MS, Niemeyer D, Goffinet C, Drosten C, Schulz S, Jäck H-M, Pöhlmann S. 2022. Evidence for an ACE2-independent entry pathway that can protect from neutralization by an antibody used for COVID-19 therapy. MBio 13:e0036422. doi:10.1128/mbio.00364-22 35467423 PMC9239067

[5] Baum A, Fulton BO, Wloga E, Copin R, Pascal KE, Russo V, Giordano S, Lanza K, Negron N, Ni M, Wei Y, Atwal GS, Murphy AJ, Stahl N, Yancopoulos GD, Kyratsous CA. 2020. Antibody cocktail to SARS-CoV-2 spike protein prevents rapid mutational escape seen with individual antibodies. Science 369:1014–1018. doi:10.1126/science.abd0831 32540904 PMC7299283

[6] Hansen J, Baum A, Pascal KE, Russo V, Giordano S, Wloga E, Fulton BO, Yan Y, Koon K, Patel K, et al.. 2020. Studies in humanized mice and convalescent humans yield a SARS-CoV-2 antibody cocktail. Science 369:1010–1014. doi:10.1126/science.abd0827 32540901 PMC7299284

[7] Lim S, Zhang M, Chang TL. 2022. ACE2-independent alternative receptors for SARS-CoV-2. Viruses 14:2535. doi:10.3390/v14112535 36423144 PMC9692829

[8] Gu Y, Cao J, Zhang X, Gao H, Wang Y, Wang J, He J, Jiang X, Zhang J, Shen G, et al.. 2022. Receptome profiling identifies KREMEN1 and ASGR1 as alternative functional receptors of SARS-CoV-2. Cell Res 32:24–37. doi:10.1038/s41422-021-00595-6 34837059 PMC8617373

[9] Yang X, Zheng X, Zhu Y, Zhao X, Liu J, Xun J, Yuan S, Chen J, Pan H, Yang J, Wang J, Liang Z, Shen X, Liang Y, Lin Q, Liang H, Li M, Peng F, Lu D, Xu J, Lu H, Jiang S, Zhao P, Zhu H. 2024. Asialoglycoprotein receptor 1 promotes SARS-CoV-2 infection of human normal hepatocytes. Signal Transduct Target Ther 9:42. doi:10.1038/s41392-024-01754-y 38355848 PMC10866945

[10] Becker S, Spiess M, Klenk HD. 1995. The asialoglycoprotein receptor is a potential liver-specific receptor for Marburg virus. J Gen Virol 76:393–399. doi:10.1099/0022-1317-76-2-393 7844558

[11] Lin G, Simmons G, Pöhlmann S, Baribaud F, Ni H, Leslie GJ, Haggarty BS, Bates P, Weissman D, Hoxie JA, Doms RW. 2003. Differential N-linked glycosylation of human immunodeficiency virus and Ebola virus envelope glycoproteins modulates interactions with DC-SIGN and DC-SIGNR. J Virol 77:1337–1346. doi:10.1128/jvi.77.2.1337-1346.2003 12502850 PMC140807

[12] Lempp FA, Soriaga LB, Montiel-Ruiz M, Benigni F, Noack J, Park YJ, Bianchi S, Walls AC, Bowen JE, Zhou J, et al.. 2021. Lectins enhance SARS-CoV-2 infection and influence neutralizing antibodies. Nature 598:342–347. doi:10.1038/s41586-021-03925-1 34464958

[13] Baggen J, Jacquemyn M, Persoons L, Vanstreels E, Pye VE, Wrobel AG, Calvaresi V, Martin SR, Roustan C, Cronin NB, Reading E, Thibaut HJ, Vercruysse T, Maes P, De Smet F, Yee A, Nivitchanyong T, Roell M, Franco-Hernandez N, Rhinn H, Mamchak AA, Ah Young-Chapon M, Brown E, Cherepanov P, Daelemans D. 2023. TMEM106B is a receptor mediating ACE2-independent SARS-CoV-2 cell entry. Cell 186:3427–3442. doi:10.1016/j.cell.2023.06.005 37421949 PMC10409496

[14] Puray-Chavez M, LaPak KM, Schrank TP, Elliott JL, Bhatt DP, Agajanian MJ, Jasuja R, Lawson DQ, Davis K, Rothlauf PW, et al.. 2021. Systematic analysis of SARS-CoV-2 infection of an ACE2-negative human airway cell. Cell Rep 36:109364. doi:10.1016/j.celrep.2021.109364 34214467 PMC8220945

[15] Riepler L, Rössler A, Falch A, Volland A, Borena W, von Laer D, Kimpel J. 2020. Comparison of four SARS-CoV-2 neutralization assays. Vaccines (Basel) 9:13. doi:10.3390/vaccines9010013 33379160 PMC7824240

[16] Schmidt F, Weisblum Y, Muecksch F, Hoffmann HH, Michailidis E, Lorenzi JCC, Mendoza P, Rutkowska M, Bednarski E, Gaebler C, Agudelo M, Cho A, Wang Z, Gazumyan A, Cipolla M, Caskey M, Robbiani DF, Nussenzweig MC, Rice CM, Hatziioannou T, Bieniasz PD. 2020. Measuring SARS-CoV-2 neutralizing antibody activity using pseudotyped and chimeric viruses. J Exp Med 217:e20201181. doi:10.1084/jem.20201181 32692348 PMC7372514

[17] Westendorf K, Zentelis S, Wang L, Foster D, Vaillancourt P, Wiggin M, Lovett E, Lee R, Hendle J, Pustilnik A, et al.. 2022. LY-CoV1404 (bebtelovimab) potently neutralizes SARS-CoV-2 variants. Cell Rep:39–110812. doi:10.1016/j.celrep.2022.110812 PMC9035363 35568025

[18] Amraei R, Yin W, Napoleon MA, Suder EL, Berrigan J, Zhao Q, Olejnik J, Chandler KB, Xia C, Feldman J, Hauser BM, Caradonna TM, Schmidt AG, Gummuluru S, Muhlberger E, Chitalia V, Costello CE, Rahimi N. 2021. CD209L/L-SIGN and CD209/DC-SIGN act as receptors for SARS-CoV-2. ACS Cent Sci 7:1156–1165. doi:10.1021/acscentsci.0c01537 34341769 PMC8265543

[19] Baggen J, Persoons L, Vanstreels E, Jansen S, Looveren D, Boeckx B, Geudens V, Man J, Jochmans D, Wauters J, Wauters E, Vanaudenaerde BM, Lambrechts D, Neyts J, Dallmeier K, Thibaut HJ, Jacquemyn M, Maes P, Daelemans D. 2021. Genome-wide CRISPR screening identifies TMEM106B as a proviral host factor for SARS-CoV-2. Nat Genet 53:435–444. doi:10.1038/s41588-021-00805-2 33686287

[20] Yan K, Dumenil T, Stewart R, Bishop CR, Tang B, Nguyen W, Suhrbier A, Rawle DJ. 2024. TMEM106B-mediated SARS-CoV-2 infection allows for robust ACE2-independent infection in vitro but not in vivo. Cell Rep 43:114921. doi:10.1016/j.celrep.2024.114921 39480813

[21] Soilleux EJ, Morris LS, Leslie G, Chehimi J, Luo Q, Levroney E, Trowsdale J, Montaner LJ, Doms RW, Weissman D, Coleman N, Lee B. 2002. Constitutive and induced expression of DC-SIGN on dendritic cell and macrophage subpopulations in situ and in vitro. J Leukoc Biol 71:445–457. doi:10.1189/jlb.71.3.445 11867682

[22] Tailleux L, Pham-Thi N, Bergeron-Lafaurie A, Herrmann J-L, Charles P, Schwartz O, Scheinmann P, Lagrange PH, de Blic J, Tazi A, Gicquel B, Neyrolles O. 2005. DC-SIGN induction in alveolar macrophages defines privileged target host cells for mycobacteria in patients with tuberculosis. PLoS Med 2:e381. doi:10.1371/journal.pmed.0020381 16279841 PMC1283365

[23] Honzke K, Obermayer B, Mache C, Fatykhova D, Kessler M, Dokel S, Wyler E, Baumgardt M, Lowa A, Hoffmann K, et al.. 2022. Human lungs show limited permissiveness for SARS-CoV-2 due to scarce ACE2 levels but virus-induced expansion of inflammatory macrophages. Eur Respir 60. doi:10.1183/13993003.02725-2021 PMC9712848 35728978

[24] Muus C, Luecken MD, Eraslan G, Sikkema L, Waghray A, Heimberg G, Kobayashi Y, Vaishnav ED, Subramanian A, Smillie C, et al.. 2021. Single-cell meta-analysis of SARS-CoV-2 entry genes across tissues and demographics. Nat Med 27:546–559. doi:10.1038/s41591-020-01227-z 33654293 PMC9469728

[25] Huot N, Planchais C, Rosenbaum P, Contreras V, Jacquelin B, Petitdemange C, Lazzerini M, Beaumont E, Orta-Resendiz A, Rey FA, Reeves RK, Le Grand R, Mouquet H, Müller-Trutwin M. 2023. SARS-CoV-2 viral persistence in lung alveolar macrophages is controlled by IFN-γ and NK cells. Nat Immunol 24:2068–2079. doi:10.1038/s41590-023-01661-4 37919524 PMC10681903

[26] Yan K, Dumenil T, Tang B, Le TT, Bishop CR, Suhrbier A, Rawle DJ. 2022. Evolution of ACE2-independent SARS-CoV-2 infection and mouse adaption after passage in cells expressing human and mouse ACE2. Virus Evol 8:veac063. doi:10.1093/ve/veac063 35919871 PMC9338707

[27] Hoffmann M, Krüger N, Schulz S, Cossmann A, Rocha C, Kempf A, Nehlmeier I, Graichen L, Moldenhauer A-S, Winkler MS, Lier M, Dopfer-Jablonka A, Jäck H-M, Behrens GMN, Pöhlmann S. 2022. The omicron variant is highly resistant against antibody-mediated neutralization: implications for control of the COVID-19 pandemic. Cell 185:447–456. doi:10.1016/j.cell.2021.12.032 35026151 PMC8702401

[28] Marzi A, Gramberg T, Simmons G, Möller P, Rennekamp AJ, Krumbiegel M, Geier M, Eisemann J, Turza N, Saunier B, Steinkasserer A, Becker S, Bates P, Hofmann H, Pöhlmann S. 2004. DC-SIGN and DC-SIGNR interact with the glycoprotein of Marburg virus and the S protein of severe acute respiratory syndrome coronavirus. J Virol 78:12090–12095. doi:10.1128/JVI.78.21.12090-12095.2004 15479853 PMC523257

[29] Brinkmann C, Hoffmann M, Lübke A, Nehlmeier I, Krämer-Kühl A, Winkler M, Pöhlmann S. 2017. The glycoprotein of vesicular stomatitis virus promotes release of virus-like particles from tetherin-positive cells. PLoS ONE 12:e0189073. doi:10.1371/journal.pone.0189073 29216247 PMC5720808

[30] Hoffmann M, Nehlmeier I, Brinkmann C, Krähling V, Behner L, Moldenhauer A-S, Krüger N, Nehls J, Schindler M, Hoenen T, Maisner A, Becker S, Pöhlmann S. 2019. Tetherin inhibits Nipah virus but not Ebola virus replication in fruit bat cells. J Virol 93:e01821-18. doi:10.1128/JVI.01821-18 30429347 PMC6340021

[31] Marzi A, Akhavan A, Simmons G, Gramberg T, Hofmann H, Bates P, Lingappa VR, Pöhlmann S. 2006. The signal peptide of the Ebolavirus glycoprotein influences interaction with the cellular lectins DC-SIGN and DC-SIGNR. J Virol 80:6305–6317. doi:10.1128/JVI.02545-05 16775318 PMC1488929

[32] Lamp B, Dietzel E, Kolesnikova L, Sauerhering L, Erbar S, Weingartl H, Maisner A. 2013. Nipah virus entry and egress from polarized epithelial cells. J Virol 87:3143–3154. doi:10.1128/JVI.02696-12 23283941 PMC3592157

[33] Sauer A-K, Liang C-H, Stech J, Peeters B, Quéré P, Schwegmann-Wessels C, Wu C-Y, Wong C-H, Herrler G. 2014. Characterization of the sialic acid binding activity of influenza A viruses using soluble variants of the H7 and H9 hemagglutinins. PLoS ONE 9:e89529. doi:10.1371/journal.pone.0089529 24586849 PMC3931807

[34] Berger Rentsch M, Zimmer G. 2011. A vesicular stomatitis virus replicon-based bioassay for the rapid and sensitive determination of multi-species type I interferon. PLoS ONE 6:e25858. doi:10.1371/journal.pone.0025858 21998709 PMC3187809

[35] Kleine-Weber H, Elzayat MT, Wang L, Graham BS, Müller MA, Drosten C, Pöhlmann S, Hoffmann M. 2019. Mutations in the spike protein of middle east respiratory syndrome coronavirus transmitted in Korea increase resistance to antibody-mediated neutralization. J Virol 93:e01381-18. doi:10.1128/JVI.01381-18 30404801 PMC6321919

